# Witnessing intimate partner violence and child maltreatment in Ugandan children: a cross-sectional survey

**DOI:** 10.1136/bmjopen-2016-013583

**Published:** 2017-02-28

**Authors:** Karen M Devries, Louise Knight, Jennifer C Child, Nambusi Kyegombe, Mazeda Hossain, Shelley Lees, Charlotte Watts, Dipak Naker

**Affiliations:** 1 Department of Global Health and Development, London School of Hygiene and Tropical Medicine, London, UK; 2 Raising Voices, Kampala, Uganda

**Keywords:** witnessing intimate partner violence, Uganda, violence against children, adolescent, physical violence, sexual violence

## Abstract

**Objectives:**

Existing evidence, mainly from high-income countries, shows children who witness intimate partner violence (IPV) at home are more likely to experience other forms of violence, but very little evidence is available from lower income countries. In this paper we aim to explore whether Ugandan children who witness IPV at home are also more likely to experience other forms of maltreatment, factors associated with witnessing and experiencing violence, and whether any increased risk comes from parents, or others outside the home.

**Design:**

A representative cross-sectional survey of primary schools.

**Participants:**

3427 non-boarding primary school students, aged about 11–14 years.

**Setting:**

Luwero District, Uganda, 2012.

**Measures:**

Exposure to child maltreatment was measured using the International Society for the Prevention of Child Abuse and Neglect Child Abuse Screening Tool-Child Institutional, and 2 questions measured witnessing IPV.

**Results:**

26% of children reported witnessing IPV, but nearly all of these children had also experienced violence themselves. Only 0.6% of boys and 1.6% of girls had witnessed partner violence and not experienced violence. Increased risk of violence was from parents and also from other perpetrators besides parents. Both girls and boys who witnessed and experienced violence had between 1.66 (95% CI 0.96 to 2.87) and 4.50 (95% CI 1.78 to 11.33) times the odds of reporting mental health difficulties, and 3.23 (95% CI 1.99 to 5.24) and 8.12 (95% CI 5.15 to 12.80) times the odds of using physical or sexual violence themselves.

**Conclusions:**

In this sample, witnessing IPV almost never occurred in isolation—almost all children who witnessed partner violence also experienced violence themselves. Our results imply that children in Uganda who are exposed to multiple forms of violence may benefit from intervention to mitigate mental health consequences and reduce use of violence. IPV prevention interventions should be considered to reduce child maltreatment. Large numbers of children also experience maltreatment in homes with no partner violence, highlighting the need for interventions to prevent child maltreatment more broadly.

**Trial registration number:**

NCT01678846, results.

**Strengths and limitations of this study**

- In high-income settings, there is a well-established link between witnessing intimate partner violence and increased risk of exposure to other violence in childhood, but there is limited evidence from low-income countries and on where increased risk of exposure to other violence is coming from.
- This paper provides rare evidence from Uganda, a low-income country, on the relationship between witnessing intimate partner violence and other forms of child maltreatment.
- We are able to explore who the perpetrators of other forms of child maltreatment are, including perpetrators both inside and outside the home.
- We also explore sequelae associated with witnessing and experiencing violence, including mental health and children's risk of using violence against others.
- The study provides valuable first evidence which may help inform intervention targeting, but is limited by its cross-sectional survey design.

## Introduction

Intimate partner violence (IPV) against women is prevalent globally, with 30% of women reporting physical and or sexual IPV in their lifetime.^1^ In addition to the known detrimental effects of IPV on women,^2^ ^3^ witnessing IPV is increasingly being recognised as an important adverse exposure for children. Effects on children include increased risk of depression, anxiety, aggression, conduct disorders, attention deficit and hyperactivity.^4^ ^5^ There is evidence that growing up in an abusive family is positively related to future violent intimate relationships.^6^ Estimates from high-income counties indicate that in the range of 8–25% of adults report exposure to IPV as children.^7^ ^8^

A growing body of research suggests that children who witness IPV are also at increased risk of being maltreated in other ways. In the USA, nationally representative data have shown that 33.9% of young people who witnessed IPV also experienced another form of maltreatment (neglect, sexual abuse by a known adult, physical abuse and psychological abuse) in the previous year, versus only 8.6% of young people who did not witness IPV. In the same study other forms of victimisation such as kidnapping, bullying and property crime was also associated with witnessing IPV.^9^ This ‘polyvictimisation’^9^ is associated with higher levels of adverse health outcomes versus single exposures.^10^

The extent of the overlap between witnessing IPV and exposure to other forms of maltreatment is not known outside high-income settings, where the epidemiology of violence exposure is likely to be quite different from the USA.^11^ Analyses often do not disaggregate by perpetrator, leaving open the question as to whether parents who have experienced or used IPV also are more likely to use violence against their children, or whether the increased risk of violence is coming from others besides parents. Additionally, studies on the health effects of witnessing IPV often do not account for the fact that witnessing may be correlated with experiencing other forms of violence.^9^ ^12–14^

We use baseline data from the Good Schools Study in Uganda to examine (1) the extent of overlap between witnessing IPV between parents, and experience of violence from other perpetrators; (2) whether witnessing is associated with increased risk of violence from parents versus perpetrators besides parents; and (3) factors associated with witnessing violence and experiencing violence. We conducted all analyses separately by sex.

## Methods

### Design

We analysed baseline survey data from the Good Schools Study,^15^ which is a cluster randomised controlled trial of the Good School Toolkit. The Toolkit was developed by Raising Voices to prevent violence against children in school and improve educational outcomes. The Toolkit is publicly available: http://raisingvoices.org/good-school/, and main trial results are available.^16^

### Setting

The survey took place in Luwero District, Uganda, from June to July 2012. Luwero is near Kampala and has both rural and urban areas.

### Sampling

We obtained a list of all 268 schools registered in Luwero in 2010 from the Ministry of Education and Sports. We excluded 97 small schools (with <40 students registered in primary 5 (P5)) and 20 schools with existing governance interventions. The remaining 151 schools formed our sampling frame. We stratified these 151 schools according to the gender ratio of pupils (>60% girls, >60% boys or about even). Forty-two schools were randomly selected, proportional to the size of the stratum. One hundred per cent of the schools agreed to participate. The sampled schools contain 79.7% of P5, P6 and P7 students in Luwero (equivalent to grades 5, 6 and 7 in the US education system; in Uganda, students in upper primary are about age 11–14 on average). Within each school, we took a simple random sample of up to 130 pupils from P5, P6 and P7 and a complete sample of school staff. If there were <130 students in a school, all were invited to participate. Seventy-seven per cent or 3706 sampled students provided data; 19% were absent from school during the week of the survey or for extended periods. The remaining 4% were entered on class lists in error, had a parent opt them out, refused or the reason for participation was not recorded. For this analysis, we excluded students who boarded at school, since their patterns of exposure to witnessing and experiencing violence from parents may differ from students who live at home.

### Procedure

For each participating school, headmasters notified staff, students and parents in advance of the survey. Parents could opt their child out of participation; otherwise individual children provided consent to participate. Data were collected in a face-to-face interview. All interviewers received 3 weeks of training on how to ask about violence in a non-judgemental way, preserve confidentiality and procedures if participants became distressed. A comprehensive child protection plan, designed by the study team in conjunction with local services, was in place to provide support to those in need of services. We also had a trained counsellor available to any child who requested counselling.

### Instruments

All items were translated into Luganda and reviewed by a panel of teachers and Raising Voices staff to ensure that they would be appropriate for Ugandan child participants and school staff. Items were then cognitively tested and refined iteratively in a sample of ∼40 children from Kampala primary schools to ensure understanding and that the meanings of original items were adequately captured. We then surveyed a larger sample of 697 children and 40 staff from Kampala schools to test distributions of items and to test study procedures.

Questions related to violence are outlined in table 1. Witnessing shouting and physical violence between parents/caregivers was measured using two binary response items developed for the study. Experiences of violence were measured by the International Society for the Prevention of Child Abuse and Neglect Child Abuse Screening Tool-Child Institutional (ICAST-CI)^17^ and some items from the WHO Multi Country Study on Women's Health and Domestic Violence against Women.^3^ Reliability and construct validity for the ICAST-CI were initially established in four countries and the instrument has since been translated into 20 languages and used extensively in multicountry research.^17^ Lifetime exposure to physical, sexual and emotional violence were constructed as binary variables. Questions related to violence were analysed by perpetrator type (parent/caretaker vs others).

**Table 1:** Definitions of violence variables

Variable name	Items	Coding
Witnessing IPV:		
Shouting	Have you ever seen or overheard your parents or caregivers shouting at each other?	Coded 1 if answered yes; 0 if answered no
Witnessing physical IPV	Have you ever seen or overheard your father hit or beat your mother?	Coded 1 if answered yes; 0 if answered no
Violence experience:	(Note: each participant asked about experience of items below, then asked about perpetrators and time frame)	
Emotional violence, (includes neglect)	Insulted you, or called you rude or hurtful names? Accused you of witchcraft? Locked you out or made you stay outside? Not given you food?	Lifetime exposure from any perpetrator. Coded 1 if answered yes to any of the items; 0 if answered no to all items
Physical violence	From perpetrators other than school staff: Twisted your arm or any other body part, slapped you, pushed you or thrown something at you? Punched you, kicked you, or hit you with a closed fist? Hit you with an object, such as a stick or a cane, or whipped you? Cut you with a sharp object or burnt you? Severe violence from school staff: Burnt you as punishment? Choked you? Tried to cut you purposefully with a sharp object? Severely beat you up?	Lifetime exposure from any perpetrator other than school staff, plus lifetime exposure to severe violence from school staff. Coded 1 if answered yes to any of the items; 0 if answered no to all items
Sexual violence	Disturbed or bothered you by making sexual comments about you? Kissed you, when you did not want them to? Touched your genitals or breasts when you did not want them to, or in a way that made you uncomfortable? Threaten or pressure you to make you do something sexual with them? Make you have sex with them, because they threatened or pressured you? Had sex with you, by physically forcing you?	Lifetime exposure from any perpetrator. Coded 1 if answered yes to any of the items; 0 if answered no to all items

IPV, intimate partner violence.

Other measures include demographic variables, use of physical and sexual violence, and mental health. Disability was measured using the following question: ‘Do you have any mental or physical disability? For example, do you have trouble seeing, walking, speaking, with fits or anything else?’ Responses were grouped into a binary variable, with students who reported any type of difficulty coded as ‘disabled’. Symptoms of common childhood mental health difficulties were measured using the Strengths and Difficulties Questionnaire (SDQ).^18^ The SDQ has been used in more than 60 different countries including several in Africa and validated in a variety of settings.^18^ In our sample, reliability for global difficulties scores was Cronbach α=0.70. The global SDQ score was constructed as a categorical variable, with children having ‘high’, ‘medium’ or ‘low’ levels of difficulties relative to their peers. To construct this measure responses to 20 items are summed, and children scoring in the highest decile of the overall distribution are deemed to have ‘high’ difficulties; the next decile to have ‘medium’ difficulties and the remaining 80% to have ‘low’ difficulties.^18^ ^19^

### Analysis

All analyses were conducted using STATA V.12.0^20^ (Intercooled Stata [program]. V.12.0. Houston, Texas: Stata Corp, 2012) and were carried out separately for male and female participants. Missing data were excluded from analyses involving those variables (pairwise deletion). In total, 3.8% of children were missing data on witnessing parents shouting, and 5.2% were missing data on witnessing physical violence.

Descriptive statistics on participants' background characteristics, witnessing violence and experiencing violence are presented by sex and compared using χ^2^ tests. When discussing results, we refer to ‘students’ or ‘boys and girls’ where findings are similar, and then present separate percentages and highlight where there are differences for boys and girls. We examined overlap between witnessing parental IPV and violence exposure from parents and perpetrators other than parents by fitting logistic regression models adjusted for a priori identified potential demographic confounders (tables 2 and 3). Next, we examined factors associated with experience of violence also, and witnessing plus experience of violence, by fitting another set of logistic regression models (tables 4 and 5).

**Table 2:** Associations between violence exposure and witnessing violence between parents, female students

Characteristic	Female (n=1423) Witnessed shouting vs witnessed no parental IPV		p Value	Female (n=1268) Witnessed physical IPV vs witnessed no parental IPV		p Value	Female (n=1364) Witnessed shouting and physical IPV vs witnessed no parental IPV		p Value
	aOR	95% CI		aOR	95% CI		aOR	95% CI	
Parental violence									
Emotional only	1.88	1.06 to 3.33	0.031	1.15	0.27 to 4.85	0.844	1.93	0.91 to 4.10	0.084
Physical only	1.51	1.05 to 2.18	0.028	1.04	0.45 to 2.41	0.930	1.74	1.09 to 2.77	0.021
Emotional and physical	3.90	1.95 to 7.81	<0.001	3.79	1.66 to 8.64	0.002	3.90	1.84 to 8.30	0.001
Non-parental violence									
Emotional	2.65	1.84 to 3.81	<0.001	1.28	0.75 to 2.21	0.356	2.02	1.37 to 2.98	0.001
Physical*	2.33	1.83 to 2.96	<0.001	1.68	1.00 to 2.81	0.049	2.54	1.82 to 3.54	<0.001
Sexual	2.36	1.49 to 3.76	0.001	3.19	0.67 to 15.14	0.139	2.98	1.81 to 4.89	<0.001

aOR is adjusted OR, adjusted for age (years), eaten at least three meals in the past day versus less, share sleeping area with or more children, versus less, share sleeping area with one or more adults versus less, have a disability versus not, worked 1–2 hours or 2 or more hours per day versus <1 hour.

*Any physical violence from non-parents but severe physical violence from school staff.

IPV, intimate partner violence.

**Table 3:** Associations between parental violence and witnessing violence between parents, male students

Characteristic	Male (n=1334) Witnessed shouting versus witnessed no parental violence			Male (n=1195) Witnessed physical IPV vs witnessed no parental violence			Male (n=1317) Witnessed shouting and physical IPV vs witnessed no parental violence		
	aOR	95% CI	p Value	aOR	95% CI	p Value	aOR	95% CI	p Value
Parental violence									
Emotional only	4.28	2.20 to 8.31	<0.001	0.74	0.08 to 6.72	0.781	6.77	3.96 to 11.56	<0.001
Physical only	1.65	0.94 to 2.89	0.077	1.05	0.36 to 3.00	0.933	0.77	0.39 to 1.51	0.436
Emotional and physical	6.53	3.29 to 12.96	<0.001	–	–		6.22	2.63 to 14.72	<0.001
Non-parental violence									
Emotional	2.67	1.86 to 3.86	<0.001	1.48	0.83 to 2.62	0.175	2.87	1.72 to 4.78	<0.001
Physical*	1.69	1.09 to 2.64	0.021	2.48	1.67 to 3.68	<0.001	2.34	1.52 to 3.56	<0.001
Sexual	1.84	0.94 to 3.61	0.074	2.24	0.57 to 8.83	0.243	3.51	1.52 to 8.10	0.004

aOR is adjusted OR, adjusted for age (years), eaten at least three meals in the past day versus less, share sleeping area with two or more children, versus less, share sleeping area with one or more adults versus less, have a disability versus not, worked 1–2 hours or 2 or more hours per day versus <1 hour.

*Any physical violence from non-parents but severe physical violence from school staff.

IPV, intimate partner violence.

**Table 4:** Factors associated with experiencing violence and experiencing violence plus witnessing IPV, female students

	Model 1 Experienced violence from any perpetrator but did not witness shouting or physical parental violence, n=1190			Model 2 Experienced violence from any perpetrator and witnessed shouting or physical parental violence, n=2477		
Characteristic	aOR	95% CI	p Value	aOR	95% CI	p Value
Age (years)	0.97	0.83 to 1.14	0.703	1.07	0.91 to 1.25	0.403
Ate at least 3 meals yesterday (vs less)	1.24	0.99 to 1.56	0.063	0.88	0.55 to 1.40	0.578
Share sleeping area with 2 or more children (vs less)	0.68	0.53 to 0.89	0.005	0.93	0.65 to 1.33	0.674
Share sleeping area with 1 or more adults (vs none)	1.03	0.73 to 1.47	0.853	1.47	0.77 to 2.83	0.239
Disability (vs not)	1.23	0.30 to 5.09	0.772	2.09	0.97 to 4.53	0.060
Work <1 hour per day	1			1		
Work 1–2 hours per day	1.52	1.08 to 2.15	0.017	1.72	1.04 to 2.86	0.036
Work more than 2 hours per day	3.69	1.83 to 7.46	0.001	4.50	1.78 to 11.33	0.002
Low SDQ score	1			1		
Medium SDQ score	2.29	1.33 to 3.95	0.004	3.84	1.69 to 8.72	0.002
High SDQ score	1.85	0.91 to 3.78	0.089	4.35	1.95 to 9.69	0.001
Used physical or sexual violence	5.14	2.99 to 8.84	<0.001	8.12	5.15 to 12.80	<0.001

aOR is adjusted OR, adjusted for all other variables in the model.

SDQ, Strengths and Difficulties Questionnaire.

**Table 5:** Factors associated with experiencing violence and experiencing violence plus witnessing, male students

Characteristic	Model 1 Experienced violence from any perpetrator but did not witness shouting or physical parental violence n=1190			Model 2 Experienced violence from any perpetrator and witnessed shouting or physical parental violence n=598		
	aOR	95% CI	p Value	aOR	95% CI	p Value
Age (years)	0.88	0.79 to 0.97	0.015	1.01	0.85 to 1.21	0.887
Ate at least 3 meals yesterday (vs less)	0.96	0.67 to 1.37	0.803	1.19	0.72 to 1.98	0.488
Share sleeping area with 2 or more children (vs less)	0.73	0.57 to 0.93	0.013	0.52	0.36 to 0.74	0.001
Share sleeping area with 1 or more adults (vs none)	1.18	0.86 to 1.63	0.301	1.16	0.68 to 1.96	0.579
Disability (vs not)	1.49	0.91 to 2.44	0.114	1.12	0.51 to 2.46	0.775
Work <1 hour per days	1			1		
Work 1–2 hours per day	1.69	1.15 to 2.49	0.008	1.86	1.14 to 3.04	0.014
Work more than 2 hours per day	1.63	0.88 to 3.02	0.119	2.07	0.91 to 4.72	0.082
Low SDQ score	1			1		
Medium SDQ score	1.66	0.96 to 2.87	0.069	2.90	1.60 to 5.26	0.001
High SDQ score	2.28	1.29 to 4.07	0.006	3.85	1.16 to 10.17	0.008
Used physical or sexual violence	3.23	1.99 to 5.24	<0.001	5.55	2.94 to 10.49	<0.001

aOR is adjusted OR, adjusted for all other variables in the model.

SDQ: Strengths and Difficulties Questionnaire.

All analyses account for the sampling scheme employed in the baseline survey—student responses are weighted to account for unequal probabilities of selection for students. SEs are adjusted for clustering at the school level using Taylor linearisation.^20^

## Results

### Demographic characteristics and prevalence of witnessing IPV

Table 6 describes the characteristics of students and prevalence of witnessing IPV in 3427 non-boarding students surveyed. The majority of students were aged 11–14 years, and more than half of all students had eaten less than three meals in the day before the survey, indicating they were possibly hungry. About 8% of the students reported some form of disability; more than 65% of students walked to school with others. Fifty-six per cent of boys but only 15% of girls indicated that they had ever worked for money, but when examining hours of paid and unpaid work on an average school day, more than half of boys and girls reported working more than 1 hour per day.

**Table 6.** Characteristics of included students

		Male			Female		
Characteristic	N*	Per cent	SE	N*	Per cent	SE	p Value
Age							<0.001
10 years or less	50	3.0	0.7	93	5.3	1.0	
11–14 years	1275	78.7	1.6	1503	84.7	1.2	
15 or more years	329	18.3	1.7	172	10.0	1.3	
Number of meals eaten yesterday							0.032
1 meal	225	12.7	1.2	280	15.1	1.2	
2 meals	716	41.7	2.0	644	36.7	1.6	
3+ meals	715	45.6	2.7	846	48.0	1.8	
Disability	122	8.0	0.7	113	7.2	0.8	0.352
Transport to school							0.019
Other	96	6.1	1.6	43	3.3	1.2	
Walking alone	443	28.7	3.1	447	26.9	3.1	
Walking with others	1067	65.3	3.3	1247	69.8	3.7	
Ever worked for money	961	55.9	3.1	272	14.7	1.2	<0.001
Hours worked on average school day							0.149
<1	570	38.2	3.8	650	38.6	2.5	
1–2	757	43.6	2.5	847	47.1	2.0	
More than 2	327	18.2	2.3	264	14.3	1.2	
Violence from parents or caregivers							
No violence	1428	87.0	1.9	1205	69.0	4.1	<0.001
Sexual violence, lifetime	0	0	–	0	0	–	
Emotional violence only, lifetime	48	2.7	0.6	78	4.4	1.3	
Physical violence only, lifetime	143	8.3	1.3	395	21.9	3.3	
Emotional and physical violence, lifetime	37	2.0	0.6	92	4.7	0.9	
Witnessing IPV							
No witnessing	1143	73.2	1.7	1199	72.9	1.6	0.836
Witnessed shouting only, ever	191	12.4	1.7	225	13.3	1.0	
Witnessed physical IPV only, ever	64	3.8	0.6	69	4.0	0.6	
Witnessed shouting and physical IPV, ever	174	10.7	1.0	165	9.8	1.0	
Violence from non-caregivers							
Emotional violence, lifetime	938	58.7	2.7	993	55.8	2.9	0.485
Physical violence,† lifetime	625	39.8	2.8	675	39.2	2.2	0.768
Sexual violence, lifetime	63	3.9	0.6	239	13.3	1.3	<0.001
Witnessing IPV‡ and violence from any perpetrator§							
Not witnessed parental IPV or experienced violence	92	5.6	0.9	72	4.6	1.6	0.446
Witnessed IPV but not experienced any violence	9	0.6	0.2	24	1.3	0.5	
Not witnessed IPV but experienced violence	1051	67.6	1.8	1127	68.3	1.6	
Witnessed IPV and experience violence	420	26.3	1.8	435	25.8	1.7	

*Ns are number of participants in each category and are not adjusted for survey design; per cent and SE are weighted and adjusted for clustering.

†Includes physical violence from any perpetrator but only severe physical violence from school staff.

‡Witnessing parental IPV includes shouting or physical IPV.

§Violence from caregiver or non-caregiver (sexual or emotional violence from any perpetrator and any physical violence from non-parents but severe physical violence from school staff).

IPV, intimate partner violence.

About 26% of boys and girls reported ever witnessing their parents shouting at each other, and ∼14% of boys and girls had ever witnessed physical violence from their mother's male partner towards their mother. Boys and girls differed dramatically on levels of violence experienced from parents or caregivers; however, 9% of girls but only 5% of boys reported emotional violence and neglect from parents or caregivers, and 27% of girls but only 10% of boys reported physical violence from parents or caregivers. No students reported sexual violence from parents or caregivers.

When examining the overlap in experience of witnessing and experiencing violence (table 6), it becomes clear that witnessing violence almost never occurs in isolation. Less than 1.3% of children reported witnessing violence without also experiencing violence themselves and 26% of boys and girls have both witnessed IPV and experienced violence from any perpetrator. About 68% of boys and 68% of girls have experienced violence but not witnessed IPV, and only 5.6% of boys and 4.6% of girls report never witnessing IPV or experiencing violence.

### What forms of violence does witnessing IPV put children at risk for?

We hypothesised that those who witnessed IPV would be at increased risk of various forms of violence from parents in particular. We found (tables 2 and 3) that girls and boys who witnessed shouting and who witnessed both shouting and physical IPV were at increased risk of emotional and combined emotional and physical violence from parents. Girls were also at increased risk for physical violence, but this association was inconsistent for boys. Boys witnessing shouting and physical IPV had over six times greater odds of experiencing emotional violence or combined emotional and physical violence from parents. Girls who witnessed shouting or shouting and physical IPV had almost four times the odds of combined emotional and physical violence compared with non-witnesses.

However, both boys and girls who witnessed IPV also had increased odds of emotional, physical and sexual violence from perpetrators *other* than parents. Children who witnessed IPV had between about 1.5 and 3.6 times the odds of reporting emotional, physical or sexual victimisation by a non-parent or caregiver, versus those who did not witness any violence between caregivers. So it appears that witnessing IPV inside the home is associated with violence both inside and outside the home.

### Factors associated with witnessing and experiencing violence

In tables 4 and 5, we examined the associations between various factors and common patterns of exposure to violence and witnessing IPV. The patterns are: experiencing violence from any perpetrator but not witnessing IPV (model 1), and witnessing IPV plus experiencing violence from any perpetrator (model 2). For both models 1 and 2, associations between demographic factors and violence or violence plus witnessing were similar. However, findings point towards additive effects of witnessing and experiencing violence on mental health and use of violence. Odds of having the higher levels of mental health difficulties and using physical or sexual violence against peers were respectively about two and five times higher in female students who experienced violence versus students who did not experience any violence (model 1). In female students who experienced and witnessed violence, the odds of having higher levels of mental health difficulties and using physical or sexual violence against peers were about four and eight times higher versus students who did not experience or witness any violence (model 2). For male students, experiencing violence was associated with about two and three times the odds of high mental health difficulties and using physical or sexual violence (model 1), whereas experiencing and witnessing violence was associated with about four and five times the odds of high levels of mental health difficulties and using physical or sexual violence (model 2).

## Discussion

In Luwero district, nearly all students who have witnessed IPV have also experienced emotional, physical or sexual violence themselves. Some of this increased risk of exposure to violence is coming from caregivers—when there is violence between caregivers, there is also likely to be violence between caregivers and children. However, children who witness IPV are also at increased risk of emotional, physical and sexual violence from other perpetrators outside the home.

The adverse effects of witnessing and experiencing violence are large. Boys and girls who have witnessed and experienced violence have nearly four and five times the odds of having high levels of mental health difficulties, and nearly six and eight times the odds of using violence, versus their boys and girls who have not experienced or witnessed violence. Evidence suggests that witnessing IPV and experiencing violence have additive effects—with children who had witnessed and experienced violence having ∼2 and 3 times the odds of mental health difficulties and using violence, respectively, compared with those experiencing violence alone.

### Other studies

In our sample, the overlap between witnessing IPV and experiencing violence was almost complete—it was extremely rare for children to witness IPV only. This differs somewhat from other representative samples from high-income settings, where witnessing is more common than exposure to maltreatment.^9^ In Uganda, similar to other countries in the region, exposure to violence from various perpetrators including parents, peers and school staff may be more normative and more chronic versus some high-income settings.^21–23^ Further research is needed to fully understand the implications of this, both in terms of the health effects of exposure, and designing appropriate intervention strategies for children in Uganda and similar settings.

In our sample, as in other samples, witnessing and experiencing IPV are strongly associated with poor mental health, and externalising behaviours such as use of violence.^24^ There are various pathways through which witnessing IPV and exposure to violence may contribute to poor mental health. Exposure to emotional, physical and sexual violence can induce a traumatic stress response, which can lead to lasting post-traumatic stress disorder, depression, anxiety and attentional and memory problems.^25^ The direct trauma and stress response of witnessing IPV itself and indirect effect on mothers mental health,^26^ disruption in caregiving due to injuries, economic effects, fathers behaviour and parenting style may all have influence on the child's mental health and well-being.^27^

In high-income settings, evidence suggests that multiple exposures to different forms of violence from different actors has an additive effect on subsequent health risks.^10^ Our evidence is consistent with this pattern, with those who are exposed to witnessing and violence showing very high odds of subsequent mental health difficulties and use of violence. Further research is needed to understand how the differing patterns of violence exposure in settings like Uganda, where some forms of violence, including corporal punishment of children, and IPV against women, may be considered more normative. The influence of context-specific norms on pathways between early violence exposure and later adverse outcomes is largely unknown.

In our sample, there is some suggestion of a sex difference in health effects, where the effects of witnessing and experiencing violence have a stronger relationship with mental health difficulties and use of violence in girls relative to boys. This may be related to the nature of the violence experienced by girls—in our sample, girls are much more likely to report sexual violence, which may have more severe effects relative to other forms of violence exposure. In the USA, witnessing partner violence is associated with a range of different forms of victimisation, but especially increases the risk of being a victim of statutory rape, sexual misconduct and dating violence.^9^ This suggests that witnessing IPV may be associated with having difficult romantic relationships in adolescence.^9^ Further work is needed to understand pathways—it could be that witnessing IPV provides a behavioural model which young people then follow when engaged in interpersonal relationships with peers and adults and in their own early romantic relationships.

It is also unclear why children who witness IPV are more likely to experience violence from other perpetrators besides parents and caregivers. Potential mechanisms could include supervision—it could be that children who live in households where parents are in a violent relationship and dysfunction is present have lower levels of supervision and parental support. In the USA, parental supervision can buffer the effects of exposure to violent environments and reduce the risk of violent victimisation for adolescents who have this parental support.^28^ We have also shown that children who witness IPV have a higher risk of mental health difficulties, which includes externalising behaviours, and have a higher risk of using violence themselves. It could be that children who have difficulties at home are more likely to behave disruptively in school and in their communities, which may increase the risk of non-caregiver adults using physical violence to punish their behaviour. Of course, there are also macrolevel factors, including socioeconomic context, poverty and related stress, and cultural and social norms that will shape risk of violence and maltreatment at the level of the family, and the community—individual experiences and behaviours must be seen in the broader socioeconomic and cultural context.

### Limitations

Although we provide some of the first data on witnessing and exposure to violence reported directly by child participants in a low-income setting, our study is not without limitations. Our data are cross-sectional; hence, we are unable to make inferences about causal relationships between witnessing violence, various forms of violence experience, mental health and other factors under study. We used a robust instrument to measure violence exposure; however, we only had two questions to measure witnessing. A more detailed set of questions may have uncovered other types of witnessing experiences which may be important for understanding health outcomes of early exposures to violence. We asked only about witnessing physical IPV from male partners to female caregivers, which may have underestimated prevalence. We also asked about violence from parents and other perpetrators in less detail relative to violence from school staff (as the main objective of our study was to document violence from school staff). This may also have underestimated prevalence. We excluded boarding students, as they may spend substantially less time at home and thus be less exposed to witnessing IPV, hence our results should not be interpreted as generalisable to this group.

In our measure of lifetime physical violence exposure, we included only severe physical violence from school staff, but both more and less severe forms of physical violence from other perpetrators. Physical violence from school staff was overwhelmingly high in our sample (more than 93% of students reported lifetime experience), hence it would have rendered our exposure measure meaningless if we had used a measure of ever exposure. Further work needs to be done to understand the relationship between different severity levels of violence from different perpetrators with health outcomes. This is a school-based sample so children not attending school, whose experience of witnessing and violence may be different, are not represented.

### Implications

Our study has shown that in our study district, homes with IPV are also highly likely to have child maltreatment. The effects of witnessing and experiencing violence on children results in poor mental health, and greatly increase the odds of use of violence by the child. Our findings suggest that interventions to reduce IPV should be explored for their efficacy in prevention of child maltreatment. One US study of maltreated children showed a decrease in internalising and externalising problems associated with resolution of IPV in the home over time.^29^

Our findings also suggest that many children are experiencing violence in homes where they are not aware of any IPV, suggesting that other child maltreatment prevention strategies are also needed. Programmes which seek to address norms and attitudes about violence against children may change levels of violence,^16^ and programmes which build safe, stable, nurturing and supportive relationships may assist children who have been maltreated or who otherwise have difficulties in achieving better outcomes.

## Conclusions

Child maltreatment and children's witnessing of IPV between caregivers overlaps substantially, and children who experience both are at greatly increased risk of poor mental health outcomes and externalising behaviour including use of violence against others. Improved understanding of the context-specific epidemiology of multiple and chronic violence exposures in settings like Uganda is needed to help develop and target interventions to reduce child maltreatment and also the adverse consequences associated with it.
